# Adherence to Congestive Heart Failure Guidelines and Outcome in the Middle East

**DOI:** 10.2174/011573403X256576231017110252

**Published:** 2023-11-23

**Authors:** Raed Aqel, Tareq Alzughayyar, Jihad Zalloum, Qais Salah, Qutaiba Qafisheh, Mahmoud Izraiq

**Affiliations:** 1 National Center for Diabetes, Endocrinology and Genetics, Amman, Jordan;; 2 College of Medicine and Health Sciences, Palestine Polytechnic University, Hebron, Palestine;; 3 Faculty of Medicine, Al Quds University, Jerusalem, Palestine;; 4 The Specialty Hospital, Private Sector, Amman, Jordan

**Keywords:** Congestive, heart failure, guidelines, Middle East, socioeconomical, ejection fraction, morbidity and mortality rates

## Abstract

**Background:**

Adherence to Congestive Heart Failure with reduced Ejection Fraction (CHFrEF) guidelines is not easily attainable everywhere, particularly in countries with a high prevalence of low socioeconomic status, which includes many Middle Eastern countries. However, it is well-established that adherence to the guidelines is associated with lower mortality and morbidity rates.

**Objective:**

Our objective is to investigate the adherence to the degree of treatment guideline in CHFrEF within a patient population in the Middle East and correlate the level of compliance both fully and partially with morbidity and mortality outcomes.

**Methods and Statistics:**

We conducted a retrospective study on patients with CHFrEF in the Middle East region who were maintained on Sacubitril/Valsartan for up to 4 years (190 patients). This study included follow-up assessments for morbidity and mortality rates and their correlation with the level of adherence to guidelines.

**Results:**

Statistical analysis was performed using IBM SPSS^®^ 27^th^ version. In both the partial adherence group and the full adherence group, there was a statistically significant improvement in NYHA (pretreatment and post-treatment) and Ejection fraction (pretreatment and post-treatment). This means that regardless of the level of adherence to the use of Sacubitril/Valsartan in CHFrEF, there was an overall improvement in the morbidity and mortality rates over the four years of follow-up.

**Conclusion:**

While we fully support the idea of achieving full CHFrEF guideline adherence, we recognize the difficulty of this task. Nevertheless, this study reinforces the notion that any degree of adherence to guideline is correlated with better morbidity and mortality rates over a long-term follow-up.

## 1 INTRODUCTION

Adherence to guideline is often linked to improved morbidity and mortality rates- a promising prospect for better health outcomes. However, the reality is far from simple. Multiple studies have revealed that strict or complete adherence to guidelines is an unfeasible task for both patients and physicians. Interestingly, institutional guidelines have been shown to influence physician adherence, which ranges from 25% to 50%, with academic institutions reporting the highest rates. Moreover, patients' adherence to guidelines is even lower, rarely surpassing 30%. This phenomenon, known as “adherence fatigue,” is a culmination of various factors, including the complexities of long-term medication use, its high costs, diverse side effects, low socioeconomical status and even physician adherence, as reflected in the time invested in patients’ education [1, 2].

The aftermath of the PARADIGM-HF and PROVE-HF studies triggered a surge of interest in the global and local efficacy of Sacubitril/Valsartan. In the Middle East, data on CHFrEF incidence, guideline application, and long-term morbidity and mortality have been rapidly accumulating. We have previously revealed eye-opening one-year follow-up data on ejection fraction improvement in Palestinian patients with CHFrEF [3]. A recent pilot study, published in 2021, offers promising results, showcasing improved morbidity and mortality rates over four years in Middle Eastern patients with CHFrEF on Sacubitril/Valsartan [4-6].

Currently, it is well-established that Sacubitril/Valsartan treatment leads to significant improvement in cardiac remodeling parameters, particularly an increase in left ventricular ejection fraction, as well as improvements in left ventricular and left atrial volumes and diastolic function [7]. These positive changes are associated with lower rates of hospitalization and mortality during follow-up.

Researchers are now exploring new areas of understanding regarding Sacubitril/Valsartan, including patients’s adherence levels and serum biomarkers and their association with clinical outcomes. For example, many experts propose a stronger association with levels of atrial natriuretic peptide (ANP), which nearly doubles in less than one month after treatment initiation. This suggests that ANP might be a more accurate marker than the inert, non-active NT-proBNP mediator in assessing treatment response and clinical outcomes [7].

In this manuscript, we seek to shine a light on the degree of adherence to guidelines for CHFrEF in the Middle East and its direct correlation with morbidity and mortality over a follow-up period of up to 4 years. This represents a new area of research, building upon our previous studies, with the hope of advancing our understanding and enhancing healthcare outcomes in the region.

## 2 METHODS

This single-center retrospective review study was conducted at a referral cardiologist clinic in the West Bank, Palestinian Territories. The data for all patients diagnosed with CHFrEF and maintained on the maximum dose of sacubitril/valsartan, following the recommendations of the Paradigm HF study [8] and the current CHFrEF guidelines [9], were collected.

All patients willingly consented to participate in this study and signed an informed consent form after being fully informed about the study protocols and objectives.

Data from all subjects' clinic visits, phone correspondences, laboratory studies, and echocardiogram results were meticulously tabulated over a follow-up period ranging from 6 months to four years.

The ejection fraction was accurately calculated by at least two board-certified cardiologists using M-Mode and/or the modified Simpson formula.

For data analysis, IBM SPSS^®^ Statistics was employed, along with the normality test and paired *T*-test. The *p*-value for NYHA and the ejection fraction before and after medication was calculated for both the full adherence and partial adherence groups.

### 2.1 Inclusion Criteria

1. Ages above 18 years.
2. Documented CHFrEF diagnosis.
3. Sacubitril/valsartan usage for at least six months between January 1, 2016, and June 30, 2019.
4. Left ventricular ejection fraction (LVEF) less than or equal to 40%.

### 2.2 Exclusion Criteria

1. Ages below 18 years.
2. LVEF greater than 40% before starting sacubitril/valsartan therapy.
3. Pregnant women.
4. Taking Sacubitril/Valsartan for less than six months.
5. Inability to obtain informed consent.

## 3 RESULTS

Among the 190 patients included in this study, follow-up was completed for 187 patients (98%), while three patients (2%) were lost to follow-up by the end of the study. A total of 156 patients (82%) were titrated to 200 mg BID of Sacubitril/valsartan, and 34 patients (18%) were titrated to 100 mg BID of sacubitril/valsartan, with follow-ups every three months until the study's conclusion. Throughout the study, patients' adherence to prescribed doses was recorded, revealing that only 38 patients (20%) adhered fully to the prescribed doses, while approximately 152 patients (80%) exhibited partial adherence, which included temporary medication holds and/or dose reductions. Notably, no patients were without any adherence, thanks to the intensive follow-up protocol. By the end of the study, 23 patients (20 from the partial adherence group (13%) and three (3%) from the full adherence group) had passed away.

IBM SPSS^®^ Statistics was utilized for data analysis, employing the normality test and paired *T*-test. The results demonstrated that the *p*-value for NYHA before and after medication delivery was 0.0001 in the partial adherence group (statistically significant) and 0.0001 in the full adherence group (also statistically significant). Similarly, the *p*-value for ejection fraction before and after medication delivery in the partial adherence group was 0.0001 (statistically significant), and 0.0009 in the full adherence group (also statistically significant).

This implies that any level of partial adherence provided a benefit in both NYHA score and EF values.

All data, including Quality of Life (QOL) described by the New York Heart Association's (NYHA) heart failure classification, laboratory data, transthoracic echocardiographic data, and mortality rate, were meticulously tabulated.

For detailed information, please refer to Tables **1A**, **1B**, **2** and **3** as well as Figs. (**1** and **2**).

## 4 DISCUSSION

Guidelines are meticulously crafted after extensive research and a thorough review of past and recent literature, all with the aim of maximizing the benefits patients can achieve. While these guidelines stress the importance of adherence to improve outcomes, it is essential to acknowledge that full adherence is not always attainable, especially in economically challenged countries where education and socioeconomic conditions may play a significant role [10, 11].

The focus of research on adherence to chronic illness treatment guidelines has largely centered around common conditions like hypertension, diabetes, and dyslipidemia. Surprisingly, close to half of the studied patients (40-65%) did not fully adhere to their prescribed medications, thereby missing out on the full benefits of their therapies [12].

Historically, poor adherence to guideline therapies has been linked to four primary factors: The patient's socioeconomic status and cultural background, the doctor-patient relationship, the patient's employment and family situation, and the complexity of the prescribed treatment [5, 11, 13].

The Qualify Registry's publication in 2017 shed light on physicians' adherence to guidelines, which ranged from 20-58%, with higher adherence observed in academic institutions. This lack of full-scale adherence is not exclusive to poorer countries; developed nations also face challenges with physician and patient adherence due to various factors [14].

Additionally, research by Rasmusoon *et al*. shows that depressive symptoms and a decline in health-related quality of life are linked to patients not taking their heart failure medications as prescribed. Health-related quality of life and depressive symptoms are two indicators that can be used to better tailor care for individuals with heart failure and thereby boost their prognosis [15].

In the Middle East, our group was among the pioneers in describing the challenges of CHFrEF, its short- and long-term follow-up, and its impact on morbidity and mortality [5]. Our pilot study revealed that achieving full adherence to guideline, even in well-established medical systems, is practically impossible. Despite taking all precautions, and implementing stringent follow-up measures, we could only achieve up to 20% full adherence. Economic burdens on patients and government budgets were among the major obstacles in our region. Additionally, common challenges, such as patient fatigue, limited education, medication side effects, lack of family support, religious beliefs influencing medical decisions, and poor physician adherence to guidelines, were prevalent [16, 17].

In our study, we questioned whether the poor adherence to CHrEF guidelines in our region would negatively affect the morbidity and mortality rates of these patients. Surprisingly and unintentionally, our findings demonstrated that any level of adherence led to improved morbidity and mortality outcomes for these patients. However, due to the small sample sizes in each medication dose group, we couldn't make direct comparisons between them. We also lacked a placebo sample, but we compensated for this limitation by comparing our mortality results with historical controls. Furthermore, we aspire to delve into the intricacies of the relationship between natriuretic peptide levels, specifically B-type natriuretic peptide (BNP) and atrial natriuretic peptide (ANP), and their direct correlation to patient adherence. Moreover, we envisage the possibility of incorporating drug-level analysis to gain a profound understanding of true adherence. Regrettably, due to the poor financial status of the majority of our patients and the high pricing of this test in our country, we find ourselves unable to embark on this ambitious endeavor. Nonetheless, we deem this new avenue of research to be of paramount importance and shall remain an eminent priority in our ongoing exploration of the therapeutic potential of sacubitril/valsartan [7, 18].

Above all, throughout the study, our intention was never to recommend partial adherence to CHFrEF guidelines. Rather, we aimed to stress the importance of full adherence while acknowledging the reality of encouraging any degree of adherence to guideline, considering the challenges faced globally and particularly in our part of the world, where legitimate reasons hinder full-scale adherence—a reality disconnect. This term refers to the realization that a world where full-scale adherence is universally achieved does not exist.

## CONCLUSION

Our pilot study highlighted the challenging nature of achieving full-scale adherence to guidelines, acknowledging that it is a difficult and ambitious task. In this reality-disconnected world, we need to be more realistic in setting our goals. Our focus should be on encouraging patients to achieve the highest level of adherence they can, recognizing that any level of adherence is valuable and can positively impact their outcomes. We should continue to motivate them to improve because every effort towards adherence matters, and it can make a significant difference in their health journey.

## Figures and Tables

**Fig. (1):**
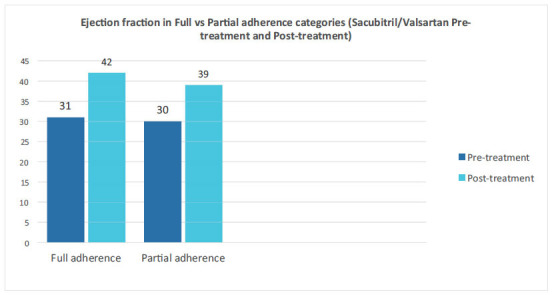
LVEF pretreatment and post-treatment in full & partial adherence groups.

**Fig. (2):**
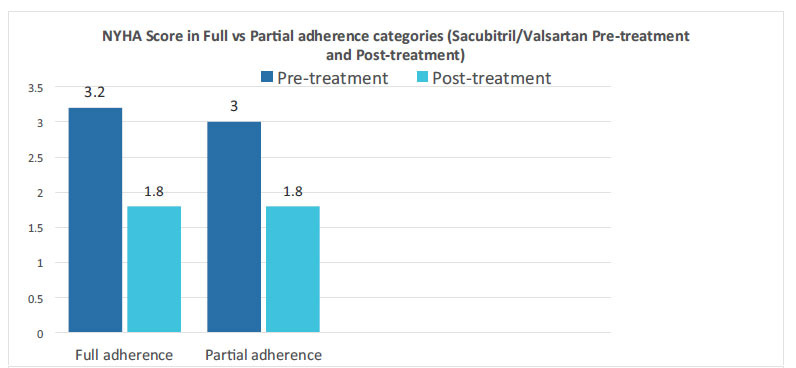
NYHA pretreatment and post-treatment in full & partial adherence groups.

**Table 1A:** Patients’ background for the full adherence group.

**Patients’ Background**	**Number of Patients**
Ischemia	24
HTN	22
DM	20
AICD	2
CRTD	1
AF	8
Smoking	14

**Table 1B:** Patients’ background for the partial adherence group.

**Patients’ Background**	**Number of Patients**
Ischemia	100
HTN	97
DM	75
AICD	16
CRTD	7
AF	21
Smoking	46

**Table 2:** Transthoracic echocardiographic data.

	**Full Adherence**		**Partial Adherence**	
	**Pre-treatment**	**Post-treatment**	**Pre-treatment**	**Post-treatment**
Left ventricular ejection fraction	31	42	30	39
New York Heart Association	3.2	1.8	3	1.8

**Table 3:** Mortality rate.

Alive	167
Deceased	23
No follow-up	3

## Data Availability

The data supporting this study's findings are openly available upon request.

## LIST OF ABBREVIATIONS

ANP: Atrial Natriuretic Peptide
QOL: Quality of Life
NYHA: New York Heart Association's
BNP: B-type Natriuretic Peptide

## STANDARDS OF REPORTING

STROBE guidelines were followed.

## References

[1] Nguyen T., Le K.K., Cao H.T.K. (2017). Association between in-hospital guideline adherence and postdischarge major adverse outcomes of patients with acute coronary syndrome in Vietnam: A prospective cohort study.. BMJ Open.

[2] DiMatteo M.R. (2004). Variations in patients’ adherence to medical recommendations: A quantitative review of 50 years of research.. Med. Care.

[3] Aqel R., Saleh M., Jubeh W. (2019). LCZ696 effect on improving quality of life and ejection fraction of Palestinian patients with heart failure and reduced ejection fraction.. J R Med Serv.

[4] Aqel R., Alzughayyar T.Z., Abukhalaf S.A., Misk R.A., Zalloum J.S. (2021). Long-term mortality and morbidity related to congestive heart failure with reduced ejection fraction (CHFrEF) in Palestinian patients maintained on submaximal sacubitril/valsartan doses: A pilot study.. J. Renin Angiotensin Aldosterone Syst..

[5] Llorca C., Cortés Castell E., Ribera Casado J.M. (2021). Factors associated with non-adherence to drugs in patients with chronic diseases who go to pharmacies in Spain.. Int J Environ.

[6] Komajda M., Schöpe J., Wagenpfeil S. (2019). Physicians’ guideline adherence is associated with long‐term heart failure mortality in outpatients with heart failure with reduced ejection fraction: The QUALIFY international registry.. Eur. J. Heart Fail..

[7] Murphy S.P., Prescott M.F., Camacho A. (2021). Atrial natriuretic peptide and treatment with sacubitril/valsartan in heart failure with reduced ejection fraction.. JACC Heart Fail..

[8] McMurray J.J.V., Packer M., Desai A.S. (2014). Angiotensin-neprilysin inhibition *versus* enalapril in heart failure.. N. Engl. J. Med..

[9] Heidenreich P.A., Bozkurt B., Aguilar D. (2022). 2022 AHA/ACC/HFSA Guideline for the management of heart failure: A report of the american college of cardiology/american heart association joint committee on clinical practice guidelines.. Circulation.

[10] Dai L., Dorje T., Gootjes J. (2023). Primary care adherence to heart failure guidelines in diagnosis, evaluation and routine management (PATHFINDER): A randomised controlled trial protocol.. BMJ Open.

[11] Komajda M., Lapuerta P., Hermans N. (2005). Adherence to guidelines is a predictor of outcome in chronic heart failure: the MAHLER survey.. Eur. Heart J..

[12] Chauke G.D., Nakwafila O., Chibi B., Sartorius B., Mashamba-Thompson T. (2022). Factors influencing poor medication adherence amongst patients with chronic disease in low-and-middle-income countries: A systematic scoping review.. Heliyon.

[13] Ahn M.S., Yoo B.S., Son J.W. (2021). Evaluation of adherence to guideline for heart failure with reduced ejection fraction in heart failure with preserved ejection fraction and with or without atrial fibrillation.. J. Korean Med. Sci..

[14] Komajda M., Cowie M.R., Tavazzi L., Ponikowski P., Anker S.D., Filippatos G.S. (2017). Physicians’ guideline adherence is associated with better prognosis in outpatients with heart failure with reduced ejection fraction: The QUALIFY international registry.. Eur. J. Heart Fail..

[15] Rasmussen A.A., Wiggers H., Jensen M. (2021). Patient-reported outcomes and medication adherence in patients with heart failure.. Eur. Heart J. Cardiovasc. Pharmacother..

[16] Parikh N.S., Parker R.M., Nurss J.R., Baker D.W., Williams M.V. (1996). Shame and health literacy: The unspoken connection.. Patient Educ. Couns..

[17] Brown M.T., Bussell J.K. (2011). Medication adherence: WHO cares?. Mayo Clin. Proc..

[18] Chen Y., Schaefer J.J., Iyer S.R. (2020). Long-term blood pressure lowering and cGMP-activating actions of the novel ANP analog MANP.. Am. J. Physiol. Regul. Integr. Comp. Physiol..

